# Unusual presentation of neurobrucellosis presenting with the features of parkinsonism: two case reports and a review of the literature

**DOI:** 10.3389/fnins.2025.1647803

**Published:** 2025-08-15

**Authors:** Moneera Aldraihem, Thamer S. Alhowaish, Yazeed Alotaibi, Mohammed AlShareet, Abdulrahman A. Alrasheed, Mazen AlAmr, Omar A. Alsinaidi, Abdulrahman S. Ali, Hisham AlDhukair

**Affiliations:** 1 Department of Neurology, National Neuroscience Institute, King Fahad Medical City, Riyadh, Saudi Arabia; 2 Department of Neurology, King Abdulaziz Medical City, Ministry of National Guard-Health Affairs, Riyadh, Saudi Arabia; 3 Prince Sultan Military Medical City, Riyadh, Saudi Arabia

**Keywords:** *brucella*, neurobrucellosis, Parkinson’s disease, parkinsonism, zoonotic disease

## Abstract

**Background:**

Brucellosis, a zoonotic disease caused by *Brucella* species, remains endemic in regions such as Saudi Arabia. While neurobrucellosis is a serious complication, its presentation with parkinsonian features and psychiatric manifestations is exceedingly rare, with only five such cases reported in the literature. These case reports add to the limited data on atypical presentations of neurobrucellosis.

**Objective:**

To describe two cases of neurobrucellosis presenting with Parkinsonism-like symptoms and psychiatric manifestations, highlighting the diagnostic challenges and therapeutic responses in a rare manifestation of this zoonotic infection.

**Design/methods:**

We report two male patients from Saudi Arabia, aged 75 and 26, diagnosed with neurobrucellosis based on high *Brucella* titers and cerebrospinal fluid polymerase chain reaction (CSF PCR) results. Both patients presented with Parkinsonism-like symptoms, including tremors, rigidity, and bradykinesia, alongside significant psychiatric disturbances such as hallucinations and cognitive impairment. Brain magnetic resonance imaging (MRI) in both cases revealed abnormalities consistent with meningoencephalitis. Both patients received a combination of antibiotics (doxycycline, rifampicin, and sulfamethoxazole-trimethoprim) and corticosteroids to manage inflammation.

**Results:**

Prolonged antibiotic therapy led to significant clinical improvement in both patients, with marked reduction in both neurological and psychiatric symptoms. Despite improvement, residual Parkinsonism-like symptoms persisted, necessitating extended therapy and long-term follow-up.

**Conclusion:**

These cases emphasize the importance of considering neurobrucellosis in patients from endemic regions with atypical neurological and psychiatric symptoms. Early diagnosis through serological and molecular testing, followed by prompt and prolonged antibiotic therapy, is crucial for favorable outcomes.

## 1 Introduction

*Brucella* is a genus of Gram-negative coccobacilli responsible for the zoonotic disease brucellosis. Recent estimates suggest that 1.6–2.1 million new human cases occur globally each year, indicating a substantial underreporting burden (Pappas et al., 2006; Laine et al., 2023). It is endemic in the Kingdom of Saudi Arabia with an estimated 214 cases per 100,000 people annually and in the Mediterranean region (Al et al., 2019). Transmission primarily occurs through the ingestion of unpasteurized dairy products; however, other routes include direct contact with infected animals, inhalation of contaminated aerosols, blood transfusion, organ or bone marrow transplantation, sexual transmission, and vertical transmission through breastfeeding (Guven et al., 2013; Yagupsky et al., 2019; Dadar et al., 2020; Tuon et al., 2017; Acharya et al., 2022).

Human brucellosis presents with highly variable severity of clinical manifestations. Common symptoms include fever, hepatosplenomegaly, arthritis, lymphadenopathy, and hematological abnormalities such as anemia, leukopenia, and lymphocytosis (Laine et al., 2023; Guven et al., 2013). Neurobrucellosis, a rare but severe complication involving the central nervous system, is predominant in 1.7–4% of all brucellosis cases. It can present at any stage of the disease with diverse neurological symptoms, including meningitis, encephalitis, myelopathy, and various neuropsychiatric manifestations (Acharya et al., 2022).

In Saudi Arabia, the prevalence of brucellosis peaked in 1990 and has since stabilized (Al et al., 2019). Studies indicate significant regional differences in the incidence within the country. Despite its diverse presentations, movement disorders caused by neurobrucellosis are exceedingly rare with few reported cases of neurobrucellosis with parkinsonism as the main neurological abnormality (Laine et al., 2023; Acharya et al., 2022; Shoaei et al., 2007; Yasin and Moghtader Mojdehi, 2014). Previous reports have documented full recovery after antibiotic treatment in such cases (Laine et al., 2023; Yasin and Moghtader Mojdehi, 2014). Here, we present two cases manifesting predominantly with parkinsonism, a rarely reported neurological presentation of neurobrucellosis, confirmed by *Brucella* serologies.

## 2 Case presentation

### 2.1 Patient 1

A 75-year-old Saudi male farmer with a history of type 2 diabetes mellitus presented with a 3-month progressive neurological decline. Initial symptoms included impaired hand coordination and task sequencing, followed by visual and auditory hallucinations. A short febrile illness with vomiting ensued, after which he developed cachexia, postural instability, frequent falls, bradykinesia, bilateral tremors, hypophonia, incontinence, and cognitive deterioration.

He denied recent travel or consumption of unpasteurized dairy products but had over 20 years of close contact with cattle, goats, and sheep.

Neurologic examination revealed inattention, non-fluent aphasia, impaired recall (MoCA 9/26), bilateral resting and kinetic tremors, lower limb rigidity, bradykinesia, and reduced strength (Medical Research Council (MRC) 3/5 right side, 2/5 left side). Hyperreflexia was present. Full sensory and cerebellar assessment was limited by his attentional deficits.

Routine labs were unremarkable except for an elevated HbA1c. *Brucella* serology was strongly positive (>1:1280), and thyroid peroxidase antibodies were also elevated. Although *Brucella* species could not be isolated by culture, the diagnosis was supported by serology and confirmed by a positive CSF *Brucella* PCR. An initial cerebrospinal fluid (CSF) analysis showed elevated protein (2.97 g/L) and mild pleocytosis (12 WBCs/mm^3^, 75% lymphocytes). A second lumbar puncture, performed approximately 2 weeks later, revealed turbid CSF, increased WBCs (14/mm^3^, 70% neutrophils), higher protein (3.37 g/L), and a slightly low glucose (4.6 mmol/L).

**Figure 1:**
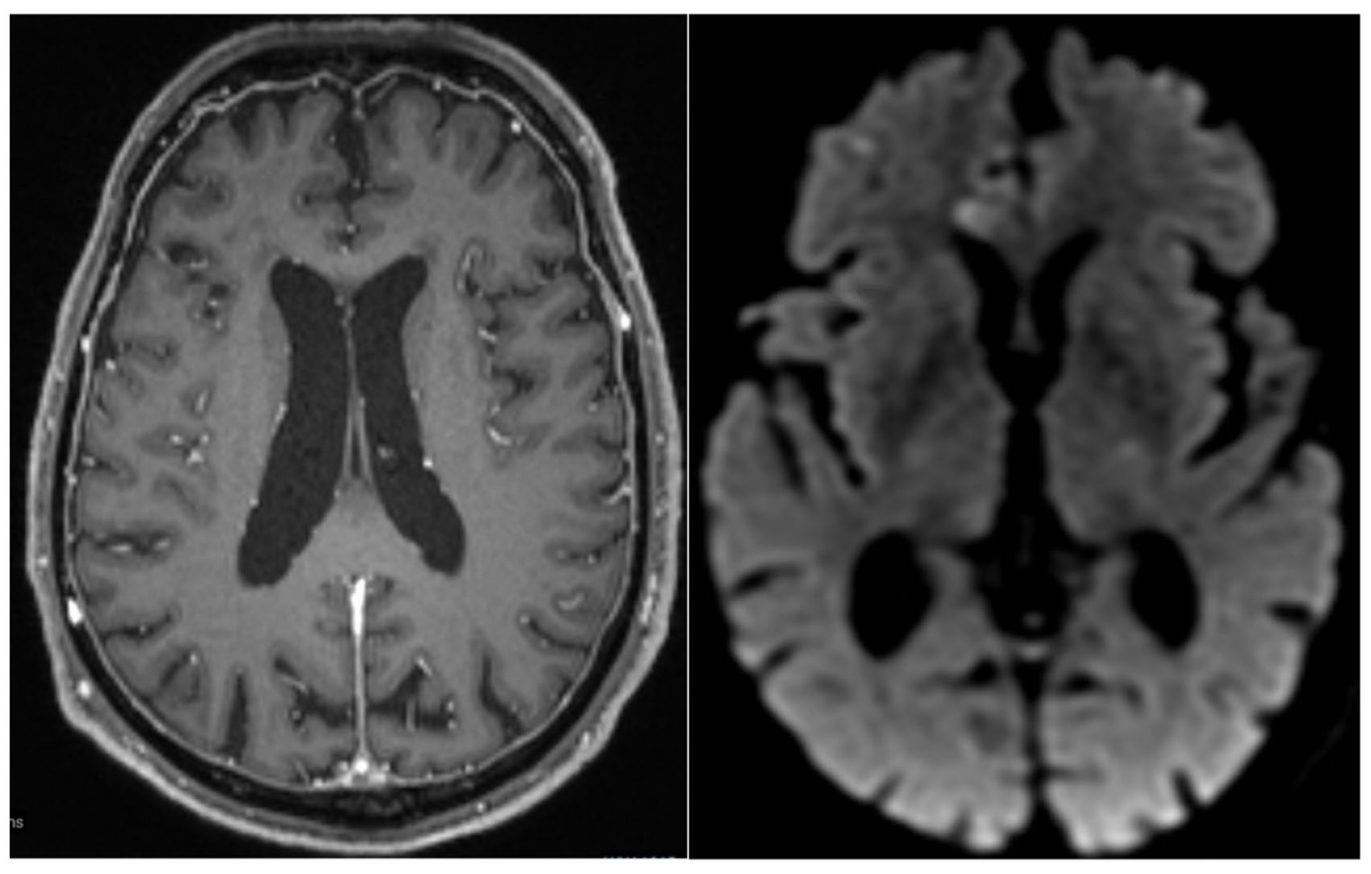
Case 1 MRI: diffuse pachymeningeal enhancement (T1 + GAD, Left); Lacunar infarcts (DWI, Right).

An initial brain MRI showed right temporal cortical lesions, acute frontoparietal lacunar infarcts, background microangiopathy, and atrophy. A second MRI, obtained 2 weeks later, demonstrated diffuse pachymeningeal and leptomeningeal enhancement (Figure 1). A third MRI, done about 2 months later, showed persistent pachymeningeal enhancement and a new focal lesion in the left temporal convexity, without acute ischemia.

He was started on a combination antibiotic regimen consisting of oral doxycycline (100 mg twice daily), oral rifampin (600 mg once daily), oral sulfamethoxazole-trimethoprim (800/160 mg twice daily), and intravenous ceftriaxone (2 g once daily, administered intermittently). This initial course continued for approximately 2.5 months. Due to persistently elevated CSF protein levels despite clinical and radiologic improvement, a second antibiotic course of the same oral regimen (doxycycline, rifampin, and sulfamethoxazole-trimethoprim) was administered for an additional 6 weeks. Corticosteroids (oral dexamethasone 4 mg every 6 h) were given concurrently during the first month and then tapered over 3 weeks in response to persistent imaging abnormalities.

CSF *Brucella* PCR became negative, and serological titers declined to 1:640, indicating microbiological response.

The patient’s clinical condition improved substantially, with resolution of hallucinations, bradykinesia, and rigidity. Strength improved to 4/5 in all limbs, though mild kinetic tremor and urinary symptoms persisted. He completed a 2-month rehabilitation program and was subsequently discharged home.

### 2.2 Patient 2

A 26-year-old Saudi male presented with a 3-week history of progressive dysarthria and unsteady gait, followed by cognitive slowing and behavioral changes such as apathy and irritability. His speech deteriorated to near mutism, coinciding with reduced interest in daily activities and episodes of anger. He reported a long-standing history of consuming raw camel milk. There was no history of fever, headache, hearing or vision loss, sensory complaints, sphincter dysfunction, weight loss, or substance use.

On examination, he was alert and oriented but showed weak eye contact, impaired short-term recall, and difficulty with abstract reasoning. Language function (comprehension, naming, reading, writing) was preserved, but speech remained severely dysarthric.

Neurological findings included saccadic intrusions, VOR overshoot, decreased visual acuity, and mild parkinsonian features—namely bradykinesia and rigidity, more prominent on the right. He also exhibited ataxia, impaired proprioception, reduced vibration and pinprick sensation, and a broad-based, imbalanced gait with reduced arm swing. Motor strength was normal.

Laboratory investigations revealed normal hematologic and metabolic profiles. ESR was elevated (63 mm/h), and CRP was mildly raised (9.4 mg/L). *Brucella* serology (BRUCELLACAPT) was markedly elevated (>1:5120). Workup for Wilson’s disease, HIV, and urine toxicology were negative. CSF analysis revealed clear fluid with:

1. RBCs: 223/mm^3^
2. WBCs: 8/mm^3^ (93% lymphocytes)
3. Protein: 0.488 g/L
4. Glucose: 3.1 mmol/L
5. CSF culture and viral PCR: Negative.

**Figure 2:**
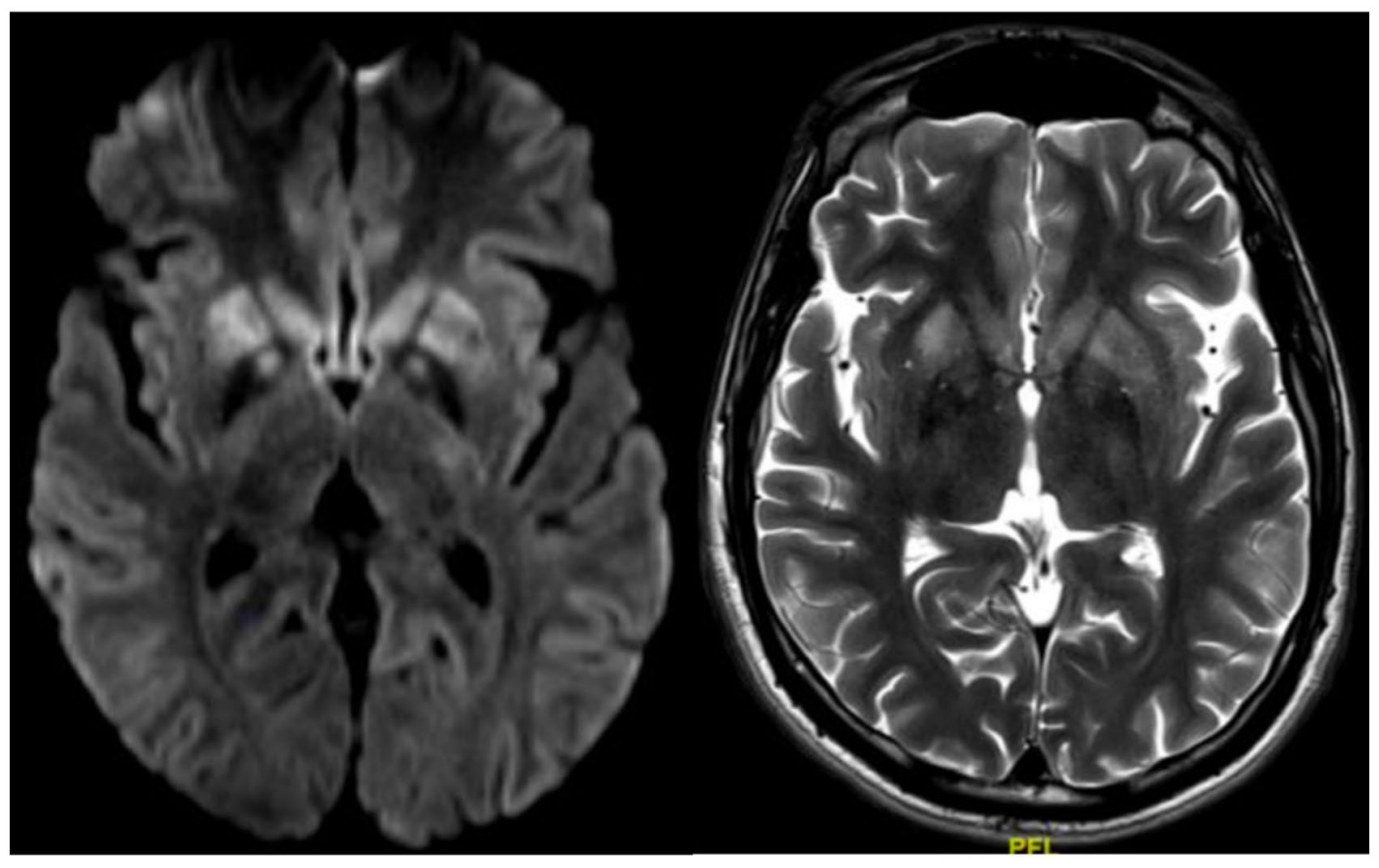
Case 2 MRI: bilateral basal ganglia and substantia nigra hyperintensities (T2, Right; DWI, Left).

Brain MRI showed extensive bilateral T2/FLAIR hyperintensities involving the basal ganglia, thalami, mammillary bodies, hippocampi, hypothalamus, optic radiations, substantia nigra, and periaqueductal gray matter, with mild restricted diffusion on DWI (Figure 2).

**Figure 3:**
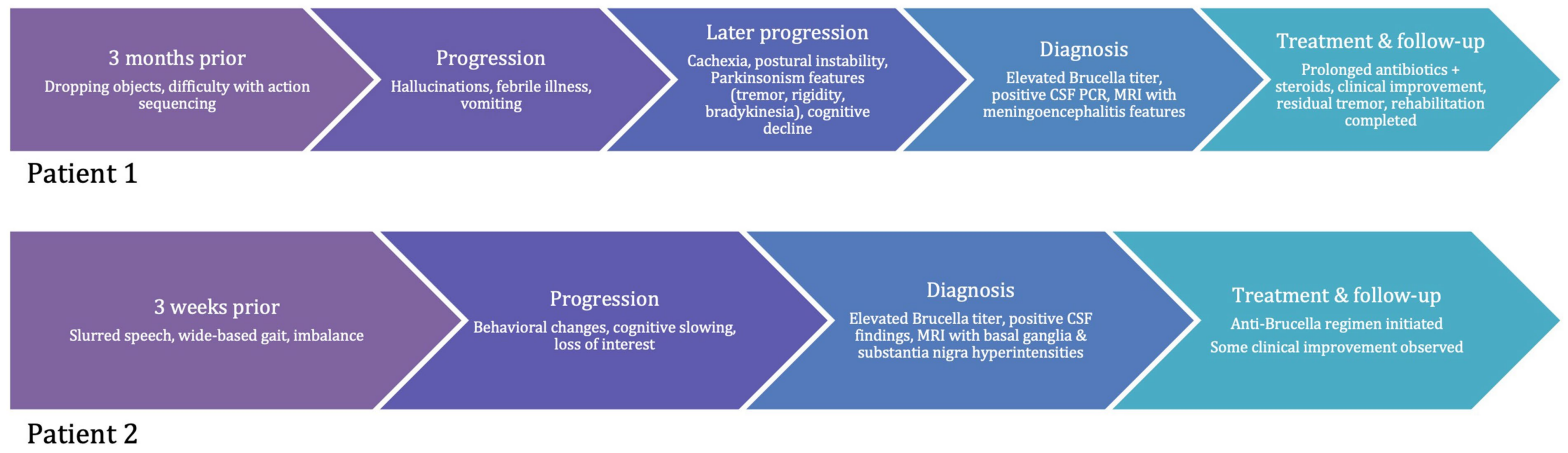
Timeline of clinical course.

The patient was started on an anti-*Brucella* regimen; however, the specific antibiotic combination, dosage, and duration were not fully documented in the medical record. On outpatient follow-up, he showed partial clinical improvement (Figure 3).

## 3 Review of reported cases of neurobrucellosis presenting with parkinsonism

### 3.1 Demographic data and clinical features

A total of five published cases of neurobrucellosis presenting with parkinsonian features have been identified in the literature. These cases are summarized in Table 1. They represent a wide demographic range, with patients aged between 23 and 78 years and include both male and female individuals, indicating no clear demographic predilection. Notably, cases were included only if the clinical description specifically mentioned tremor, bradykinesia, rigidity, or other parkinsonism-related signs, in keeping with the neurological syndrome of interest.

**Table 1:** Summary of reported cases of neurobrucellosis presenting with parkinsonism.

Case report	Patient demographics	Clinical presentation	Diagnostic tests	Treatment	Outcome
1987 BMJ case report (Molins et al., 1987)	68-year-old woman	Slow gait, tremors, muscle rigidity	Positive *Brucella melitensis* blood cultures, CSF normal	Doxycycline, streptomycin, rifampin	Symptom resolution
2007 Iranian case report (Shoaei et al., 2007)	23-year-old male	Tremor, muscle rigidity, meningovascular involvement	Positive Wright test, lymphocytic pleocytosis in CSF	Ceftriaxone, rifampin, doxycycline, cotrimoxazole	Developed bilateral hearing loss, transient hemiparesis
2008 Iranian case report (Ghaffarinejad et al., 2008)	26-year-old male	Psychosis, parkinsonian symptoms	Positive Wright test and 2-ME test, MRI showing T2 bright signals	Doxycycline, rifampin, cotrimoxazole, risperidone	Decreased frequency of psychotic symptoms, parkinsonian symptoms subsided
2013 case report from Jiangsu Province Hospital (Jin et al., 2013)	52-year-old male	Parkinsonian-like tremor, motor seizures	Positive Rose Bengal Plate and Standard Tube Agglutination tests	Trimethoprim/sulfamethoxazole, levofloxacin	Symptom resolution without anti-Parkinson drugs
2014 Iranian case report (Yasin and Moghtader Mojdehi, 2014)	78-year-old man	Acute parkinsonism, hyponatremia due to SIADH	Positive serology, CSF analysis	Doxycycline, rifampin, ceftriaxone	Full recovery

### 3.2 Clinical features

All five cases exhibited parkinsonian motor symptoms, though the severity and combination of features varied. Tremor was a consistent finding, ranging from resting tremor to parkinsonian-like tremors that were static or stress-exacerbated. For example, Jin et al. described a patient with a parkinsonian-like tremor without other classical features of Parkinson’s disease (Jin et al., 2013), while Yasin et al. reported a more complete parkinsonian syndrome with tremor, rigidity, and bradykinesia (Yasin and Moghtader Mojdehi, 2014). Other cases, such as those reported by Shoaei et al. and Molins et al., also documented classic parkinsonian rigidity and slow gait (Shoaei et al., 2007; Molins et al., 1987).

In addition to parkinsonism, three patients presented with cognitive or psychiatric manifestations, such as hallucinations, behavioral changes, or frontal lobe dysfunction. The 2008 Iranian case described by Shoaei et al. involved a 26-year-old male with psychosis and parkinsonian symptoms (Ghaffarinejad et al., 2008). Focal neurological deficits, including hemiparesis, were noted in two patients (Shoaei et al., 2007; Yasin and Moghtader Mojdehi, 2014), and seizures were reported in one case (Jin et al., 2013).

### 3.3 Investigations

All cases underwent confirmatory diagnostic workup. Serologic testing for *Brucella* (e.g., Rose Bengal, Wright, and 2-ME tests) was positive in all five cases. CSF analysis revealed lymphocytic pleocytosis and elevated protein in most cases. Although CSF culture results were often negative, these were complemented by positive serology or other microbiological evidence.

MRI findings included white matter hyperintensities, basal ganglia T2 changes, and meningeal enhancement in some patients (Shoaei et al., 2007; Yasin and Moghtader Mojdehi, 2014; Ghaffarinejad et al., 2008). EEG, when performed, showed nonspecific slowing (Jin et al., 2013; Ghaffarinejad et al., 2008). These variable findings illustrate the need for high clinical suspicion and a comprehensive diagnostic approach, especially in endemic regions.

### 3.4 Treatment

All patients received multidrug antibiotic regimens, typically including doxycycline, rifampin, ceftriaxone, or trimethoprim-sulfamethoxazole. Treatment durations were prolonged, reflecting the intracellular persistence of *Brucella* spp. Corticosteroids were added in selected cases to control inflammatory complications.

### 3.5 Prognosis

Clinical outcomes were generally favorable with appropriate therapy. Most patients showed resolution or significant improvement of parkinsonian symptoms. However, residual deficits such as hearing loss or behavioral changes persisted in some cases (Shoaei et al., 2007). These findings reinforce the importance of early recognition and sustained treatment (Shoaei et al., 2007; Ghaffarinejad et al., 2008).

## 4 Discussion

Brucellosis is one of the most prevalent zoonotic infections globally, with an estimated 500,000 new human cases reported annually, though more recent data suggest this figure may be significantly higher (Pappas et al., 2006; Laine et al., 2023). The disease is endemic in areas where livestock farming is common, including the Mediterranean, the Middle East, Central Asia, and parts of Latin America and Africa. In Saudi Arabia, the incidence remains relatively high, estimated at 214 cases per 100,000 people annually, with regional variability (Al et al., 2019). Neurobrucellosis, a rare but potentially severe complication of brucellosis, occurs in approximately 3–5% of cases, typically affecting the central nervous system through hematogenous dissemination (Laine et al., 2023; Guven et al., 2013).

Neurobrucellosis is clinically heterogeneous and can present with meningitis, meningoencephalitis, cranial nerve involvement, myelitis, psychiatric symptoms, or, more rarely, movement disorders. In a cohort of 39 patients with neurobrucellosis, Guven et al. reported headache (76.9%), fever (66.7%), vomiting (43.6%), and meningeal signs (43.6%) as the most common symptoms. Neurological complications included cranial neuropathies (35.9%), myelopathy (10.3%), and psychiatric symptoms (10.3%) (Guven et al., 2013).

Our two cases broaden this clinical spectrum. Both patients exhibited parkinsonian features—an exceedingly rare presentation of neurobrucellosis—alongside cognitive and behavioral disturbances. Tremor was reported in most, but not all, reviewed cases, highlighting variability in motor presentations across the literature. These findings add to the limited literature describing extrapyramidal involvement in brucellosis-related CNS infection.

A key diagnostic clue in both cases was the epidemiological exposure history. The first patient had long-standing occupational exposure to goats and sheep, while the second reported regular consumption of raw camel milk—both well-documented risk factors for *Brucella* transmission (Yagupsky et al., 2019; Dadar et al., 2020). These details underscore the critical role of thorough history-taking and risk factor assessment, particularly in endemic regions, to avoid delays in diagnosis.

Diagnosis was confirmed through CSF analysis and positive *Brucella* PCR, supporting the value of molecular tools when culture results are negative. Both patients received prolonged antimicrobial therapy, consisting of doxycycline, rifampin, sulfamethoxazole-trimethoprim, and ceftriaxone. Corticosteroids were added in the setting of radiological inflammation. Both patients showed significant neurological improvement, though mild residual deficits persisted.

These cases emphasize that neurobrucellosis should remain in the differential diagnosis of parkinsonism and neuropsychiatric presentations in endemic areas. Awareness of atypical features, coupled with targeted diagnostics and early treatment, is essential for improving outcomes.

## 5 Conclusion

These cases highlight the importance of considering neurobrucellosis in patients from endemic areas presenting with atypical neurological symptoms. High clinical suspicion, combined with appropriate diagnostic tests, is essential for timely diagnosis and management. These cases contribute to the growing body of literature on neurobrucellosis, emphasizing its varied presentations and the importance of tailored antibiotic therapy for successful outcomes.

## Data Availability

The original contributions presented in the study are included in the article/supplementary material, further inquiries can be directed to the corresponding author.
