# Correction: Unusual presentation of neurobrucellosis presenting with the features of parkinsonism: two case reports and a review of the literature

**DOI:** 10.3389/fnins.2026.1804126

**Published:** 2026-02-20

**Authors:** Moneera Aldraihem, Thamer S. Alhowaish, Yazeed Alotaibi, Mohammed AlShareet, Abdulrahman A. Alrasheed, Mazen AlAmr, Omar A. Alsinaidi, Abdulrahman S. Ali, Hisham AlDhukair

**Affiliations:** 1 Department of Neurology, National Neuroscience Institute, King Fahad Medical City, Riyadh, Saudi Arabia; 2 Department of Neurology, King Abdulaziz Medical City, Ministry of National Guard-Health Affairs, Riyadh, Saudi Arabia; 3 Prince Sultan Military Medical City, Riyadh, Saudi Arabia

**Keywords:** *Brucella*, neurobrucellosis, Parkinson's disease, parkinsonism, zoonotic disease

Author Abdulrahman A. Alrasheed was erroneously assigned to Affiliation 1 (Department of Neurology, National Neuroscience Institute, King Fahad Medical City, Riyadh, Saudi Arabia).

The correct affiliation for Abdulrahman A. Alrasheed is Affiliation 3: Prince Sultan Military Medical City, Riyadh, Saudi Arabia.

The original version of this article has been updated.

